# An Ancestral Mechanism of Calmodulin Binding to Cds1 Kinase Inhibits Catalytic Activity

**DOI:** 10.1002/iub.70128

**Published:** 2026-08-26

**Authors:** Stephanie A. Manovella, Tingting Wang, Samuel N. Young, Toby A. Dite, Vineet Vaibhav, Steve Binos, Laura F. Dagley, Anh T. N. Nguyen, Anthony R. Means, Janni Petersen, John W. Scott, James M. Murphy, Christopher R. Horne

**Affiliations:** ^1^ Drug Discovery Biology Monash Institute of Pharmaceutical Sciences, Monash University Melbourne Victoria Australia; ^2^ Walter and Eliza Hall Institute of Medical Research Parkville Victoria Australia; ^3^ Flinders Health and Medical Research Institute, Flinders University Adelaide South Australia Australia; ^4^ Department of Medical Biology University of Melbourne Parkville Victoria Australia; ^5^ Molecular and Cellular Biology Baylor College of Medicine Houston Texas USA

## Abstract

Calmodulin is a highly conserved, calcium (Ca^2+^) sensor protein that is ubiquitous among eukaryotes. Ca^2+^ binding to Calmodulin induces a conformational change that facilitates interaction with, and activation of, serine/threonine protein kinases, including members of the CaMK family. Recently, Ca^2+^‐Calmodulin binding to one such protein kinase, Checkpoint kinase 2 (CHK2), which is responsible for the regulation of cell cycle progression following DNA damage in mammalian cells, was shown to suppress CHK2 catalytic activity. Here, by applying biochemical, structural mass spectrometry and yeast genetic methods, we identify an analogous mode of inhibition of the fission yeast *Schizosaccharomyces pombe* CHK2 functional orthologue, Cds1, through direct binding of Ca^2+^‐Calmodulin to the Cds1 kinase domain. Our studies assert an ancestral function for Calmodulin in suppressing the catalytic activity of CHK2 orthologs and highlight a mechanism by which Ca^2+^ flux can attenuate Cds1 catalytic activity to facilitate exit from the replication checkpoint and promote cell cycle progression.

## 1 Introduction

Calcium (Ca^2+^) is an essential second messenger in eukaryotic cellular signalling, which serves crucial functions in controlling cell motility, gene transcription, muscle contraction and exocytosis [1]. Ca^2+^ levels within cells are spatiotemporally controlled by multiple mechanisms, including extracellular Ca^2+^ influx via transmembrane ion channels and Ca^2+^ pumps, and release from intracellular Ca^2+^ storage organelles [2]. Subsequently, elevations in Ca^2+^ concentrations are detected by sensor proteins, such as Calmodulin, which undergo conformational changes upon Ca^2+^ binding to license their interaction with, and modulation of, partner proteins [3].

Calmodulin is a highly conserved and ubiquitous Ca^2+^‐sensing protein among eukaryotes [4] with a broad repertoire of protein interactors, which in humans totals in excess of 300 targets [5]. Mammalian Calmodulin is a single 149‐amino‐acid polypeptide chain that adopts a dumb‐bell like structure, where the N‐ and C‐terminal EF‐hand domains each bind two Ca^2+^ [3, 4, 6]. Binding of Ca^2+^ to the C‐terminal EF‐hand domain [7] triggers a conformational change that increases the binding affinity of Ca^2+^ to the N‐terminal EF‐hand domain to which Ca^2+^ binding subsequently exposes a hydrophobic pocket within the domain, allowing Calmodulin to engage with target proteins [8].

The repertoire of proteins that bind to and are regulated by Ca^2+^‐Calmodulin includes protein kinases, many of which are members of the Ca^2+^‐Calmodulin‐dependent protein kinase (CAMK) family of serine/threonine protein kinases [9]. Canonically, Calmodulin binding to a motif that overlaps an autoinhibitory sequence C‐terminal to the kinase domain facilitates kinase activation. However, not all CAMK family members harbour a canonical Calmodulin‐binding sequence, which compromises prediction of which kinases might be Calmodulin‐regulated. Recent findings suggest additional complexity, such that Calmodulin binding to the kinase domain itself, rather than peripheral sequences, can either promote or negate kinase activity [10, 11, 12, 13]. One case where Calmodulin binding suppresses kinase activity is the highly‐studied CAMK family protein kinase, Checkpoint kinase 2 (CHK2), which is essential for regulating cell cycle progression following DNA damage [11, 14].

A functional homologue of the mammalian checkpoint kinase CHK2 is the protein kinase Cds1, a crucial effector of the replication checkpoint in fission yeast, *Schizosaccharomyces pombe* [15]. Cell cycle checkpoints function as regulatory surveillance mechanisms that prevent the cell from progressing to the next stage of the cell cycle until all critical processes, such as DNA replication and mitosis, are complete [16]. *Cds1* deletion, kinase‐dead knock‐in, or overexpression in *S. pombe* each lead to halted cell division upon exposure to replicative stress [17, 18, 19, 20, 21]. Following replicative stress, an absence of Cds1 or Cds1 catalytic activity precludes a G2/M cell cycle block, leading to premature entry into mitosis despite incomplete DNA replication or damage repair, ultimately leading to cell death. Elevated Cds1 activity drives expression of Mik1 kinase and activation of Wee1 kinase [18, 19], each of which can phosphorylate Cdc2 kinase at Y15 to inhibit Cdc2 activity and halt replication checkpoint exit [22]. Cds1 and another kinase, Chk1, which is a crucial effector in the double‐stranded break/DNA repair response, coordinately phosphorylate Cdc25 phosphatase on S99 [20], leading to Cdc25 sequestration via 14‐3‐3 binding [21] and incapacity to dephosphorylate Cdc2, thus preventing cell cycle progression [19].

Because Ca^2+^‐Calmodulin binding suppressed the activity of the mammalian Cds1 homologue, CHK2 [11], we sought to determine whether such a regulatory mechanism might be ancestrally conserved. Indeed, here we report that Ca^2+^‐Calmodulin exerts an inhibitory effect on Cds1 kinase activity via direct engagement of the Cds1 kinase domain. Chemical crosslinking mass spectrometry (XL‐MS) confirmed that Ca^2+^‐Calmodulin binds to the kinase domain of Cds1, as well as the FHA domain, consistent with AlphaFold modelling and analogous to observations reported for human CHK2. Additionally, we report that relief of Ca^2+^‐Calmodulin suppression of Cds1 catalytic activity through mutagenic disruption of the Calmodulin binding interface imbalances the Cds1‐Chk1 kinase equilibrium, leading to suppression of yeast proliferation. Overall, our data support an ancestral mechanism of Calmodulin binding to Cds1 kinase to inhibit catalytic activity, allowing cells to exit the replication checkpoint and proceed through the S to M phase of the cell cycle.

## 2 Materials and Methods

### 2.1 Expression Constructs

The gene encoding full‐length *Schizosaccharomyces pombe* Cds1 (Uniprot Q09170) was synthesised by GenScript (New Jersey, USA) and subcloned into the insect expression vector, pFastBac GST, as an in‐frame fusion with a TEV protease‐cleavable N‐terminal GST tag using the BamHI and EcoRI restriction sites. A variant encoding K317A Cds1 was generated by oligonucleotide‐directed overlap PCR mutagenesis using oligonucleotides synthesised by IDT (Singapore). The gene encoding full‐length *S. pombe* Calmodulin (cam1) (Uniprot P05933) was synthesised by IDT (Singapore) as a gBlock and subcloned into the bacterial expression vector, pPROEX Htb (Life Technologies), as an in‐frame fusion with a TEV protease‐cleavable N‐terminal hexahistidine tag. Insert sequences were verified by Sanger sequencing (AGRF, VIC, Australia).

### 2.2 Recombinant Expression and Purification

Full‐length Cds1 was expressed and purified from Expi*Sf*9 insect cells using established procedures [10, 11, 23]. Briefly, bacmids encoding wild‐type or K317A Cds1 were prepared following transformation and recombination in DH10MultiBac *E. coli* (ATG Biosynthetics). Expi*Sf*9 insect cells were cultured in Expi*Sf* CD media (Thermo Fisher Scientific). Each bacmid (1 μg) was introduced into 0.9 × 10^6^ Expi*Sf*9 cells by Cellfectin II (Thermo Fisher Scientific) mediated transfection in six‐well plates using the Bac‐to‐Bac protocol (Thermo Fisher Scientific). After 4 days of static incubation at 27°C in a humidified incubator, the resulting P1 baculovirus was harvested and added to 50 mL Expi*Sf*9 cells at 1.0 × 10^6^ cells/mL density at 2% v/v, which were shaken at 27°C, 130 rpm. The cell density was monitored daily using a haemocytometer slide and maintained at 0.5–3.0 × 10^6^ cells/mL by diluting with fresh Expi*Sf* CD media when necessary, until growth arrest (defined as a cell density less than the twice the cell count 1 day prior). Approximately 24 h after growth arrest was recorded, the P2 baculovirus was harvested by collecting the supernatant after pelleting the cells at 500 × *g* for 5 min. P2 baculovirus was added to 0.5 L Expi*Sf* cells cultured at 4.5–5.5 × 10^6^ cells/mL in 2.8 L Fernbach flasks at 0.6% v/v, and cultured at 27°C, 90 rpm for 72 h. Cells were harvested at 500 × *g* and pellets snap frozen in liquid N_2_ and either thawed immediately for lysis or stored at −80°C.

Cds1 cell pellets were resuspended in GST buffer (20 mM HEPES pH 7.5, 200 mM NaCl, 10% v/v glycerol), supplemented with cOmplete Protease Inhibitor (Roche) and 0.5 mM Bond‐Breaker TCEP [tris‐(2‐carboxyethyl)phosphine] (Thermo Fisher Scientific) and were lysed by sonication before the lysate was clarified by centrifugation (40,000 × *g*, 45 min, 4°C). The proteins were maintained at 4°C throughout purification. The clarified lysate was incubated with Glutathione Xpure Agarose resin (UBP‐Bio) pre‐equilibrated in GST buffer for > 1 h on rollers at 4°C, before the beads were pelleted at 500 × *g* and washed extensively with GST buffer. Cds1 was cleaved from the GST tag (on resin) with recombinant His_6_‐TEV protease, and incubated with His‐Lambda Phosphatase (both made in‐house) and 2 mM MnCl_2_ overnight at 4°C, followed by addition of Ni‐NTA resin (cOmplete His‐Tag; Roche) to eliminate TEV protease and Lambda Phosphatase. The eluate was spin concentrated (30 kDa MWCO; Millipore) before being loaded onto a Superdex 200 10/300 GL (Cytiva) pre‐equilibrated with SEC buffer (20 mM HEPES pH 7.5, 200 mM NaCl, 5% v/v glycerol). Purified fractions, as assessed following resolution by reducing StainFree SDS‐PAGE gel electrophoresis (Bio‐Rad), were pooled and spin concentrated to 5 mg/mL based on A_280_ and extinction coefficient, aliquoted, and snap frozen in liquid N_2_ for storage at −80°C.

*S. pombe* Calmodulin was expressed in *E. coli* BL21‐CodonPlus‐RIL (Agilent) cells cultured in Super Broth supplemented with ampicillin (100 μg/mL) at 37°C with shaking at 220 rpm to an OD600 of ~0.6–0.8. Protein expression was induced by the addition of isopropyl β‐D‐1‐thiogalactopyranoside (IPTG; 500 μM) and the temperature was lowered to 18°C for expression overnight. Cells were harvested at 500 × *g* and pellets snap frozen in liquid N_2_ and either thawed immediately for lysis or stored at −80°C. Calmodulin cell pellets were resuspended in Ni‐NTA buffer (20 mM HEPES pH 7.5, 200 mM NaCl, 10% v/v glycerol), supplemented with 10 mM imidazole (pH 8.0), EDTA‐free cOmplete Protease Inhibitor (Roche) and 0.5 mM Bond‐Breaker TCEP, and lysed by sonication before the lysate was clarified by centrifugation (40,000 × *g*, 45 min, 4°C). The clarified lysate was incubated with Ni‐NTA resin (Roche) pre‐equilibrated in Ni‐NTA buffer with 5 mM imidazole for > 1 h on rollers at 4°C, before the beads were pelleted at 500 × *g* and washed extensively with Ni‐NTA buffer containing 35 mM imidazole. The proteins were eluted in Ni‐NTA buffer containing 500 mM imidazole. The His_6_ tag was cleaved using recombinant His_6_‐TEV protease and dialysis performed in SEC buffer (20 mM HEPES pH 7.5, 200 mM NaCl, 5% v/v glycerol), supplemented with 10 mM EGTA pH 7.5. The dialysate was further purified by the addition of Ni‐NTA resin (cOmplete His‐Tag; Roche) to eliminate uncleaved material and the TEV protease. The eluate was then spin concentrated (10 kDa MWCO; Millipore) and supplemented with 10 mM EGTA (Ca^2+^ chelator) before being loaded onto a Superdex 75 10/300 GL (Cytiva) pre‐equilibrated with SEC buffer. Purified fractions, as assessed following resolution by reducing StainFree SDS‐PAGE gel electrophoresis (Bio‐Rad), were pooled and spin concentrated to 30 mg/mL based on A_280_ and extinction coefficient, aliquoted, and snap frozen in liquid N_2_ for storage at −80°C. The protein purity of each construct is shown in Supplementary Figure 1a.

### 2.3 Intact Mass Spectrometry

Purified recombinant wild‐type and K317A Cds1, and Calmodulin, (~10 μg) were resolved by reverse‐phase chromatography on a 25 cm ProSwift RP‐4H monolith column (Thermo Scientific) using a micro‐flow HPLC (Ultimate 3000). The HPLC was coupled to a Maxis II Q‐TOF mass spectrometer (Bruker) equipped with an ESI source. Peptides were loaded directly onto the column for online buffer exchange at a constant flow rate of 50 μL/min with buffer A (99.9% Milli‐Q water, 0.1% FA) using a valve to direct flow to waste. After 10 min, the valve was switched to direct flow to the mass spectrometer, and peptides were eluted with a 10 min linear gradient from 2% to 90% buffer B (99.9% ACN, 0.1% FA). The Maxis II Q‐TOF was operated in full MS mode using Compass Hystar 6.2. Settings for intact samples run per day method were as follows: Mass Range 100 to 3000 m/z, Capillary Voltage 4500 V, Dry Gas 4 L/min, Dry Temp 200°C. Data was analysed with Data Analysis version 6.1 (Bruker) and peptide masses were deconvoluted using the maximum entropy method. Data are shown in Supplementary Figure 1b–d.

### 2.4 Radiometric Cds1 Kinase Assay

Cds1 activity was determined by measuring the transfer of radiolabelled phosphate from [γ‐^32^P]‐ATP to a synthetic peptide substrate (CHKtide; KKKVSRSGLYRSPSMPENLNRPR), synthesised by GenScript (New Jersey, USA). Briefly, purified recombinant *S. pombe* Cds1 (50 ng) was incubated in assay buffer (50 mM HEPES, pH 7.4, 1 mM DTT) containing 200 μM CHKtide, 1 mM CaCl_2_, 200 μM [γ‐^32^P]‐ATP (Perkin Elmer, MA, USA), 5 mM MgCl_2_ (Sigma) and up to 50 μM recombinant *S. pombe* Calmodulin in a standard 25 μL assay for 10 min at 30°C. Reactions were terminated by spotting 15 μL onto phosphocellulose paper (SVI‐P; St Vincent's Institute, Melbourne) and washing extensively in 1% phosphoric acid (Sigma). Radioactivity was quantified by liquid scintillation counting. Additional experiments were carried out using an analogous reaction (as above) with titrations of Calmodulin (50, 25, 12.5, 6.25, 3.13, 1.56, 0.78, 0.19, 0.01, 0 μM) and CaCl_2_ (10,000, 5000, 2500, 1250, 625, 310, 78, 39, 20, 10, 0 μM) to assess dose dependence. GraphPad Prism 10 was used for data analysis.

### 2.5 Thermal Stability Assays

Thermal stability experiments were performed using a Tycho NT.6 differential scanning fluorimeter (NanoTemper Technologies). Purified recombinant wild‐type and K317A Cds1 (~30 μg in SEC buffer) were loaded into Tycho NT.6 glass capillaries (Cat# TY‐C001) and placed in the instrument. Thermal scans were conducted from 35°C to 95°C at a scan rate of 0.5°C s^−1^ with a 285 nm excitation wavelength. Fluorescence intensity was measured at 0.1°C intervals at emission wavelengths of 330 and 350 nm. The first derivative of the 350/330 nm ratio was used to calculate the melting temperature (*T*_m_) of each sample in °C. Each protein was analysed in two independent experiments. *T*_m_ values are reported as mean ± standard deviation.

### 2.6 Chemical Crosslinking Sample Preparation

For 4‐(4,6‐dimethoxy‐1,3,5‐triazin‐2‐yl)‐4‐methylmorpholinium chloride (DMTMM) crosslinking, full‐length recombinant *S. pombe* Cds1 and Calmodulin (1:5 molar ratio) were initially incubated on ice in 50 mM HEPES (pH 7.5), 10 mM CaCl_2_ for 30 min. The protein mixture (~3 μg) was then incubated for 30 min at room temperature with a range of DMTMM concentrations (0, 5, 10, 20, 50 and 100 mM; Sigma, Cat# 74104), which was prepared in 50 mM HEPES (pH 7.5). Following incubation, each reaction was quenched with 100 mM Tris–HCl (pH 7.5), mixed with reducing sample buffer, boiled at 100°C for 5 min, then resolved by reducing SDS‐PAGE gel electrophoresis (Bio‐Rad). SDS‐PAGE gels were stained using SimplyBlue SafeStain (Thermo Fisher Scientific). For SDA crosslinking, full‐length recombinant *S. pombe* Cds1 and Calmodulin (1:2 molar ratio) were initially incubated on ice in 50 mM HEPES (pH 7.5), 10 mM CaCl_2_ for 30 min. Protein (1 mg/mL) was then mixed with SDA (NHS‐Diazirine; 1 mg/mL; Thermo Fisher Scientific, Cat# 26167) and incubated in the dark for 30 min at room temperature to react the NHS‐ester group. The diazarine group was then photo‐activated using ultraviolet light irradiation (UVP CL‐1000 L UV cross‐linker) at 365 nm. Samples were added to upturned lids excised from 1.5 mL microfuge tubes and placed on ice at a distance of 5 cm from the lamp and irradiated for 1 min. The reaction mixtures from the titration were combined and quenched with 100 mM Tris–HCl (pH 7.5), mixed with reducing sample buffer, boiled at 100°C for 5 min, then resolved by reducing SDS‐PAGE gel electrophoresis (Bio‐Rad). SDS‐PAGE gels were stained using SimplyBlue SafeStain (Thermo Fisher Scientific) and crosslinked adducts excised for mass spectrometry analysis (based on their migration relative to the MW marker).

### 2.7 Mass Spectrometry Sample Processing

Excised protein gel bands were destained using 50% acetonitrile for 30 min at 37°C. The gel band was then dehydrated by adding 100% acetonitrile (ACN) and incubating at room temperature for 10 min before aspirating the ACN and further drying the gel piece with the vacuum centrifuge (CentriVap, Labconco). Proteins were reduced by the addition of 1 mM dithiothreitol (DTT) in 50 mM ammonium bicarbonate for 30 min. Excess DTT was aspirated before the addition of 55 mM iodoacetamide to alkylate the sample for 60 min at room temperature. The gel slice was then washed with 50% acetonitrile twice and 100% ACN once before drying to completion in the vacuum centrifuge. Proteins were digested overnight with 500 ng trypsin in 50 mM ammonium bicarbonate at 37°C and extracted the following day using 60% acetonitrile/0.1% formic acid. The collected peptides were lyophilized to dryness using a CentriVap (Labconco) before reconstituting in 10 μL 0.1% formic acid/2% ACN ready for mass spectrometry analysis.

Reconstituted peptides were analyzed on Orbitrap Eclipse Tribrid mass spectrometer interfaced with Neo Vanquish liquid chromatography system. Samples were loaded onto a C18 fused silica column (inner diameter 75 μm, OD 360 μm × 15 cm length, 1.6 μm C18 beads) packed into an emitter tip (IonOpticks) using pressure‐controlled loading with a maximum pressure of 1500 bar using a Nanoflex source and electrosprayed directly into the mass spectrometer.

We first employed a linear gradient of 3%–30% of solvent‐B at 400 nL/min flow rate (solvent‐B: 99.9% (v/v) ACN) for 100 min, followed by a gradient of 30%–40% solvent‐B for 20 min and 35%–99% solvent‐B for 5 min. The column was then maintained at 99% B for 10 min before being washed with 3% solvent‐B for another 10 min comprising a total of 145 min run with a 120 min gradient in a data dependent (DDA) mode. MS1 spectra were acquired in the Orbitrap (*R* = 120 k; normalised AGC target = standard; MaxIT = Auto; RF Lens = 30%; scan range = 380–1400; profile data). Dynamic exclusion was employed for 30 s excluding all charge states for a given precursor. Data dependent MS2 spectra were collected in the Orbitrap for precursors with charge states 3–8 (*R* = 50 k; HCD collision energy mode = assisted; normalised HCD collision energies = 25%, 30%; scan range mode = normal; normalised AGC target = 200%; MaxIT = 150 ms).

### 2.8 Mass Spectrometry Data Analyses

Raw data files were converted to MGF files using MS convert [24]. For DMTMM crosslinking analysis, peak files were searched using the MeroX software [25] (version 2.0.1.4) and a FASTA file containing the full‐length *S. pombe* Cds1 and Calmodulin sequences. K to D or E residues were set as specificity sites for DMTMM crosslinking (—H_2_O). Trypsin was set as the enzyme allowing for three missed cleavages. Maximum peptide length was set to 40. Precursor precision was set at 10 ppm with fragment ion precision set at 20 ppm. For SDA crosslinking, MGF files were searched against a FASTA file containing the *S. pombe* Cds1 and Calmodulin sequences using XiSearch software [26] (version 1.8.13) with the following settings: crosslinker = multiple, SDA and noncovalent; first linked amino acid (any), second linked amino acid (KSTY, N‐term); fixed modifications = Carbamidomethylation (C); variable modifications = oxidation (M), SDA (KSTY, N‐term) DELTAMASS: 110.04801284, SDA‐loop (KSTY, N‐term) DELTAMASS: 82.04186484, SDA‐hydro (KSTY, N‐term) DELTAMASS: 100.052430; MS1 tolerance = 3.0 ppm, MS2 tolerance = 6.0 ppm; losses = H_2_O, NH_3_, CH_3_SOH; CleavableCrossLinkerPeptide:MASS: 82.04186484. False Discovery Rate (FDR) analysis was performed with the in‐built xiFDR set to 5%. Data were visualised using the xiVIEW software [27]. For SDA crosslinking, MGF files were also re‐searched as above with the inclusion of phosphorylation of (Ser, Thr & Tyr) as a variable modification.

### 2.9 Computational Modelling of the Cds1:Calmodulin Complex

The full‐length sequences of *S. pombe* Cds1 (Uniprot Q09170) and *S. pombe* Calmodulin (cam1; Uniprot P05933) were used to generate a structural model of the Cds1:Calmodulin complex, in the presence of four Ca^2+^ ions, using AlphaFold3 [28]. The confidence metrics are shown in Supplementary Figure 2.

### 2.10 Yeast Cell Culture

All cultures were grown at 28°C and cultured in log phase for 48 h. Cells were inoculated in Yeast Extract Supplemented (YES) based media [29]. To induce genotoxic stress, hydroxyurea was added to YES agar plates at a final concentration of 2 mM. All cell proliferation assays (colony‐forming assays) were performed with cells from early exponential cultures 1.2 × 10^6^ cells/mL.

### 2.11 Generation of Cds1 Lysine/Arginine to Alanine Mutations

The *Cds1* gene (650 bp upstream and 450 bp downstream of the open reading frame) was amplified by PCR from genomic DNA and cloned into the pCRTM‐Blunt II‐TOPO vector (Thermo Fisher Scientific). Cds1 lysine (K202, K317) and arginine (R412) to alanine point mutations were generated by site‐directed mutagenesis. Transformation of a *Cds1*::*ura4*+ deletion and selection on YES‐FOA plates [29] were used to select for integration of *Cds1* K202A, K317A and R412A point mutations into the yeast genome.

### 2.12 Semi Quantifications of Yeast Proliferation

Proliferation relative to wild type cells; the ability to form colonies on agar plates on which serial dilution of cells from liquid cultures were spotted was quantified from brightness values using ImageJ. GraphPad Prism 10 was used for data analysis. Unpaired *t*‐tests were used for all semi quantifications of growth. A 95% confidence interval was used for calculating significance.

## 3 Results

### 3.1 Calmodulin Allosterically Inhibits Cds1 Catalytic Activity

**FIGURE 1:**
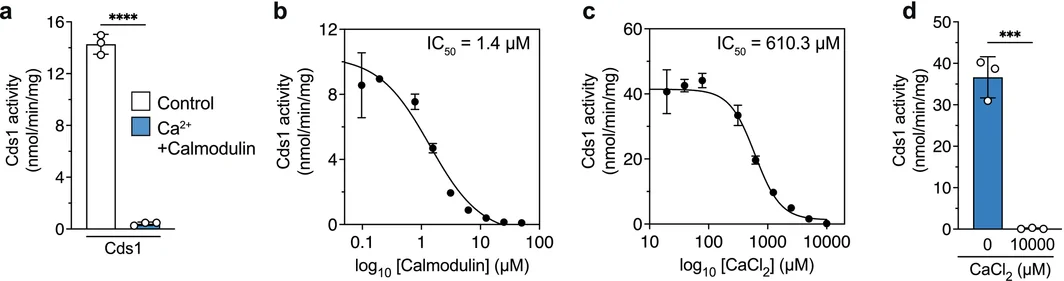
Cds1 activity is inhibited by increased levels of Calcium and Calmodulin. (a) Radiometric assay of full‐length wild‐type Cds1 expressed in insect cells with Ca^2+^‐Calmodulin. Cds1 showed robust catalytic activity in the absence of Calmodulin (white) when Ca^2+^ was present (1 mM), while in the presence of Ca^2+^ (1 mM) and Calmodulin (50 μM), Cds1 activity was inhibited by ≥ 90% (blue). Individual data points are shown as circles with accompanying mean ± SD; *n* = 3. Statistical analysis was performed by unpaired t‐test; ****p < 0.0001. (b) Cds1 catalytic activity was assayed across a range of Calmodulin concentrations (50, 25, 12.5, 6.25, 3.13, 1.56, 0.78, 0.19, 0.01, 0 μM) in the presence of 10 mM Ca^2+^. The half‐maximal inhibitory concentration (IC_50_) of Calmodulin was 1.4 μM. Statistical analysis was performed with *n* = 3 by using the non‐linear regression equation [inhibitor] versus response (three‐parameter fit). (c) Cds1 catalytic activity was assayed across a range of Ca^2+^ concentrations (10,000, 5000, 2500, 1250, 625, 310, 78, 39, 20, 10, 0 μM) in the presence of 50 μM Calmodulin. The half‐maximal inhibitory concentration (IC_50_) of Ca^2+^ was 610.3 μM. Statistical analysis was performed with *n* = 3 by using the non‐linear regression equation [inhibitor] versus response (four‐parameter fit). (d) Plot of highest and lowest Cds1 kinase activity from the CaCl_2_ titration, both in the presence of 50 μM Calmodulin. High concentrations of CaCl_2_ (10 mM) inhibited Cds1 activity, whereas no CaCl_2_ did not alter Cds1 activity. Individual data points are shown as circles with accompanying mean ± SD; *n* = 3. Statistical analysis was performed by unpaired t‐test; ***p < 0.001.

Upon Ca^2+^ binding, we recently found that Calmodulin directly binds to human CHK2 via its kinase domain to inhibit catalytic activity [11]. To investigate whether this mode of allosteric regulation by Calmodulin is evolutionarily conserved, we performed radiometric kinase assays using recombinant full‐length Cds1, measuring the transfer of radiolabelled phosphate from [γ‐^32^P]‐ATP to the synthetic peptide CHKtide. Similar to human CHK2 suppression by human Calmodulin [11], we found that *S. pombe* Calmodulin inhibited Cds1 activity by ≥ 90% (Figure 1a; Supplementary Figure 1). To further validate these results and determine the IC_50_, which serves as a surrogate for binding affinity of the interaction, we titrated Calmodulin up to 50 μM in our radiometric assay. These titrations gave a half‐maximal inhibition (IC_50_) value for Cds1 by *S. pombe* Calmodulin of 1.4 μM (Figure 1b), analogous to the low micromolar binding affinity reported for human Calmodulin towards human CHK2 [11]. Next, we sought to determine the Ca^2+^ dependence of Calmodulin with Cds1 by titrating CaCl_2_ up to 10 mM in our radiometric assay. These titrations showed an IC_50_ of Cds1 by Calmodulin at 610.3 μM Ca^2+^ (Figure 1c,d), which, albeit at high micromolar, falls within a physiologically relevant range for eukaryotic cells, including yeast (10^−7^ to 10^−6^ M) [30, 31]. Critically, neither Calmodulin nor Ca^2+^ alone affected Cds1 activity, underscoring the importance of Ca^2+^ in promoting a conformational state of Calmodulin that enables Cds1 recognition and inhibition.

### 3.2 Calmodulin Directly Binds the FHA and Kinase Domains of Cds1

**FIGURE 2:**
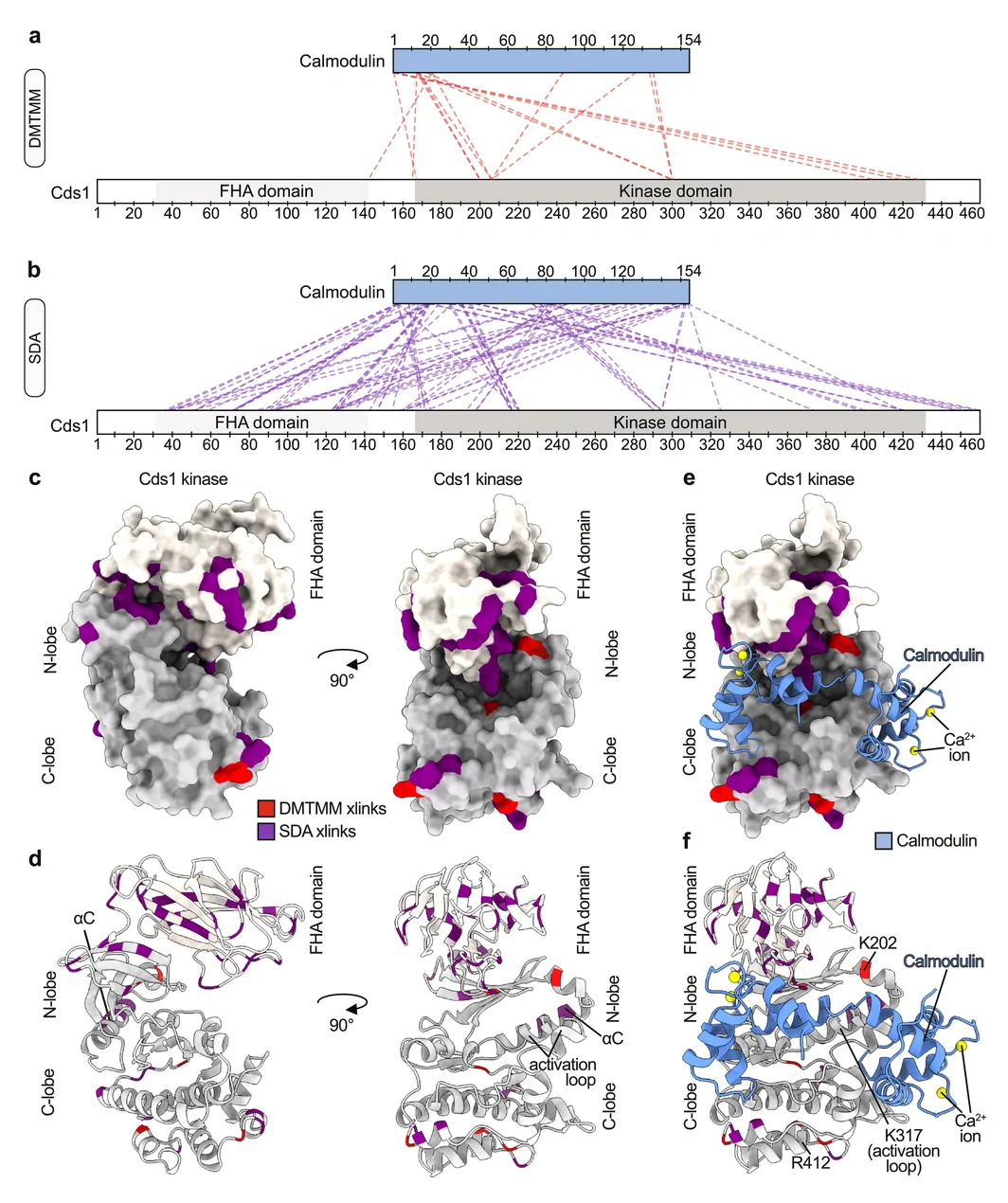
Calmodulin inhibits Cds1 activity through the FHA and kinase domain. (a) DMTMM chemical crosslinking on Cds1 and Calmodulin analysed by mass spectroscopy. DMTMM cross‐linking Calmodulin (blue) with Cds1 (grey). Red dashed lines represent crosslinks. (b) SDA chemical cross‐linking Calmodulin (blue) with Cds1 (grey). Purple dashed lines represent crosslinks. (c) AlphaFold surface model of Cds1 kinase domain (grey). FHA domain is highlighted in off‐white. DMTMM and SDA crosslinks are shown in red and purple, respectively. (d) Richardson (Ribbons) diagram of an AlphaFold model of the Cds1 kinase domain (grey). FHA domain is highlighted in off‐white. DMTMM and SDA crosslinks are shown in red and purple, respectively. (e) AlphaFold model of the Cds1 kinase domain (surface) and Calmodulin (ribbon; blue). FHA domain is highlighted in off‐white. DMTMM and SDA crosslinks are shown in red and purple, respectively. Ca^2+^ ions are shown as yellow spheres. (f) AlphaFold ribbon model of the Cds1 kinase domain (grey) and Calmodulin (blue). FHA domain is highlighted in off‐white. DMTMM and SDA crosslinks are shown in red and purple, respectively. Evolutionary conserved residues for Calmodulin binding (K202, K317 and R412) are highlighted. Ca^2+^ ions are shown as yellow spheres.

Having established a direct inhibitory interaction between Cds1 and Ca^2+^‐Calmodulin, we next sought to define their specific binding interfaces using mass spectrometry with the zero‐length crosslinker, DMTMM (Figure 2a) and the photoactivatable crosslinker, succinimidyl 4,4′‐azipentanoate (SDA; also known as NHS‐Diazirine) (Figure 2b). Using full‐length recombinant Cds1 with either crosslinker, we observed multiple crosslinks with Calmodulin that were localised to the Cds1 FHA and kinase domains. The bulk of DMTMM‐mediated crosslinks to Calmodulin mapped to a surface centred on the kinase domain within a Cds1 AlphaFold model (Figure 2a,c), while SDA yielded additional crosslinks between Calmodulin and the FHA domain of Cds1 (Figure 2b,c). Notably, fewer SDA crosslinks were detected when searches included phosphosites (28 vs. 81), although the same overall pattern of crosslinks was observed, in addition to phosphorylation within the kinase domain at Y193, T213 and T291 (Supplementary Figure 3). The crosslinks observed in both SDA and DMTMM experiments were consistent with interactions predicted in an AlphaFold model of Calmodulin bound to Cds1. In this model, the dumbbell‐like structure of Calmodulin is positioned face‐to‐face with the Cds1 kinase domain, encompassing both lobes of the kinase domain, centred on the regulatory αC helix and catalytic loop at the interlobe cleft, with proximity to the FHA domain (Figure 2d). Experimental crosslinks provide support for the modelled Cds1:Calmodulin complex; however, low pLDDT scores were obtained for the complex model, especially within both the FHA domain of Cds1 and Calmodulin, indicative of likely high protein flexibility [32]. Calmodulin is known to be conformationally malleable [33], which, along with the micromolar affinity for Calmodulin for Cds1 (Figure 1b), would be expected to introduce dynamicity into the Cds1 and Calmodulin interaction.

### 3.3 Cds1 K317 Is Indispensable for *S. Pombe* Proliferation Under Genotoxic Stress

**FIGURE 3:**
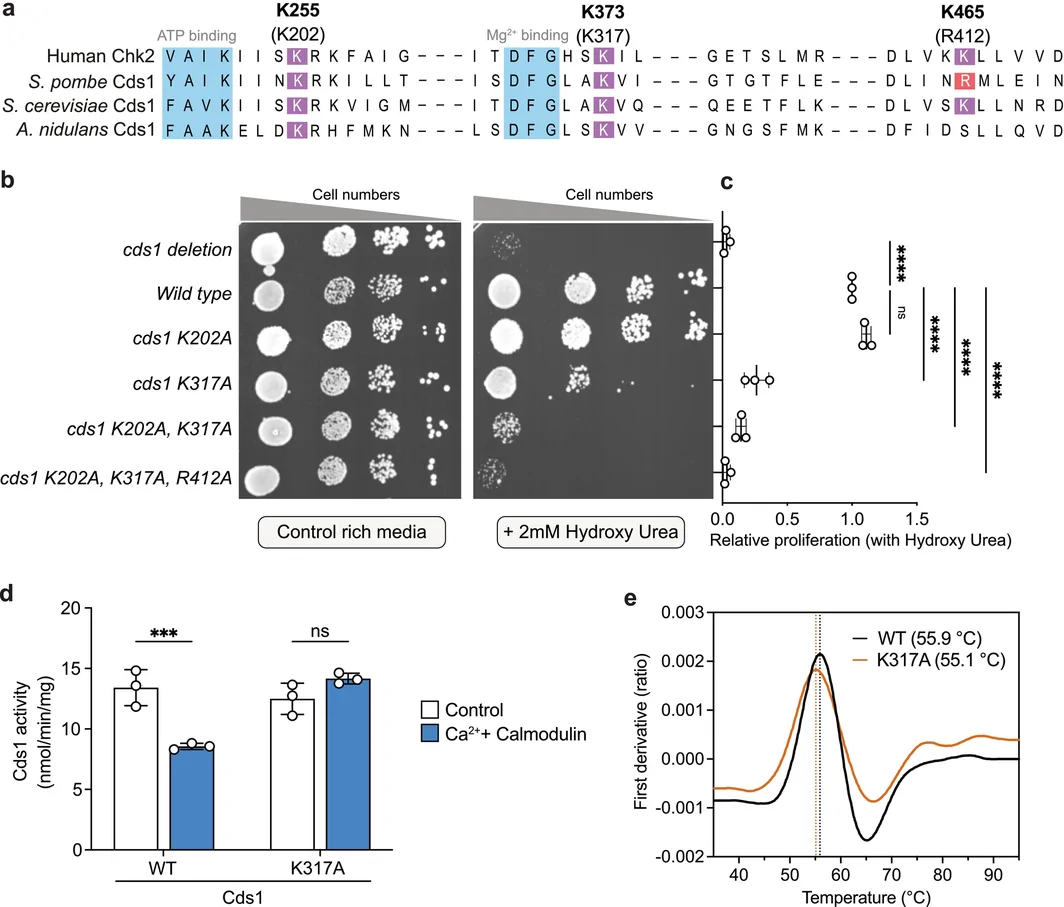
Key residues in Cds1 are evolutionarily conserved and play a critical role in supporting cell proliferation. (a) Comparison of human CHK2 sequences implicated in Calmodulin binding by chemical crosslinking experiments [11] (top), and *S. pombe* Cds1, *Saccharomyces cerevisiae* Cds1 and *Aspergillus nidulans* Cds1 counterpart sequences. ATP and Mg^2+^ binding motifs are highlighted in blue. Conserved lysine and arginine residues of interest are highlighted purple and red, respectively. Human CHK2 residues are shown in bold above the alignment, while the *S. pombe* counterpart residues are shown in plain text within brackets. (b) *S. pombe* proliferation in controlled rich media (left) or 2 mM hydroxyurea to instigate a genotoxic environment (right) identifies important roles of the conserved lysine and arginine residues in cell proliferation following cellular stress. (c) Quantitation of *S. pombe* proliferation for wild‐type and mutant Cds1, as well as Cds1 deletion following exposure to hydroxyurea. Individual data points are shown as circles with accompanying mean ± SD; *n* = 3. Statistical analysis was performed by one‐way ANOVA; ****p < 0.0001; ns = not significant. (d) Radiometric assay of recombinant wild‐type and K317A Cds1 in the presence of 1 mM Ca^2+^ with (blue) or without (white) Calmodulin (50 μM). In the presence of Ca^2+^‐Calmodulin (50 μM), wild‐type Cds1 activity was significantly inhibited, whereas K317A Cds1 activity remained unchanged, which supports a loss of sensitivity to Ca^2+^‐Calmodulin inhibition. Individual data points are shown as circles with accompanying mean ± SD; *n =* 3. Statistical analysis was performed by two‐way ANOVA; ***p < 0.001; ns = not significant. (e) Thermal stability of wild‐type (black line; 55.9°C ± 0.20) and K317A (gold line; 55.1°C ± 0.17) Cds1, measured by nano differential scanning fluorimetry. Melting curves are representative of two independent measurements, where the T_m_ is marked with a dashed vertical line.

We next sought to determine whether evolutionarily conserved residues in human CHK2 and *S. pombe* Cds1, which reside at the centre of the modelled Calmodulin‐Cds1 interaction, were important for cell proliferation under genotoxic stress. Previously, our crosslinking mass spectrometry studies identified K373 within the human CHK2 activation loop as a central site for Calmodulin binding and catalytic activity attenuation [11] Sequence comparisons between CHK2 and Cds1 identified the counterpart of CHK2 K373, Cds1 K317, in addition to other modelled interface residues, K202 and R412, as conserved between species (Figure 3a). In addition to Cds1 K317 lying at the heart of the Calmodulin interaction validated using crosslinking‐mass spectrometry, we observed crosslinks between Cds1 K202 and Calmodulin in DMTMM experiments (Figure 2), providing further support for an ancestral interaction mode. Accordingly, we used gene editing to introduce alanine substitutions of each residue in *S. pombe* and examined the impact of these mutations on growth in cell proliferation assays. Cds1 mutants and deletion strains were generated by substituting Lys or Arg with Ala using site‐directed mutagenesis and homologous recombination with a *ura4+* gene. Under normal conditions, wild‐type Cds1 and each mutant or deletion strain showed comparable growth (Figure 3b, left panel). However, as Cds1 is a critical factor in the replication checkpoint, we repeated these experiments in the presence of hydroxyurea to induce genotoxic stress. Cell proliferation in the presence of hydroxyurea was significantly inhibited in strains harbouring the K317A, K202A/K317A, and K202A/K317A/R412A Cds1 mutations, as well as in the *Cds1* deletion strain (Figure 3b,c). Cell proliferation has been used previously to probe Cds1 function in *S. pombe* [17], providing a robust and quantifiable measure of signalling perturbations that are known to reflect other phenomena, such as the genotoxic stress‐induced DNA damage termed ‘cut’. Notably, K317A mutant Cds1 exhibited catalytic activity comparable to wild‐type Cds1 in our radiometric assay (Figure 3d; Supplementary Figure 1) but was immune to inhibition by Ca^2+^‐Calmodulin, in keeping with K317 serving an evolutionarily conserved function as a site of Calmodulin action to dampen Cds1 activity.

The lack of availability of suitable antibodies towards Cds1 precluded analysis of the relative expression level of wild‐type and K317A Cds1 in *S. pombe*. However, thermal stability assays performed on recombinant full‐length wild‐type and K317A Cds1 indicated that the introduction of the mutation did not compromise Cds1 stability (Figure 3e). Therefore, we anticipate that K317A Cds1 is likely to be expressed comparably to the wild‐type counterpart in *S. pombe*. Our finding that the mutation of Cds1 K317 to alanine compromises cell cycle progression, as reflected by reduced viability in the presence of hydroxyurea, illustrates the essential role of Calmodulin‐mediated attenuation of Cds1 activity, and highlights the delicate balance between Cds1 and Chk1 activities in governing the response to cellular stress and DNA damage.

## 4 Discussion

**FIGURE 4:**
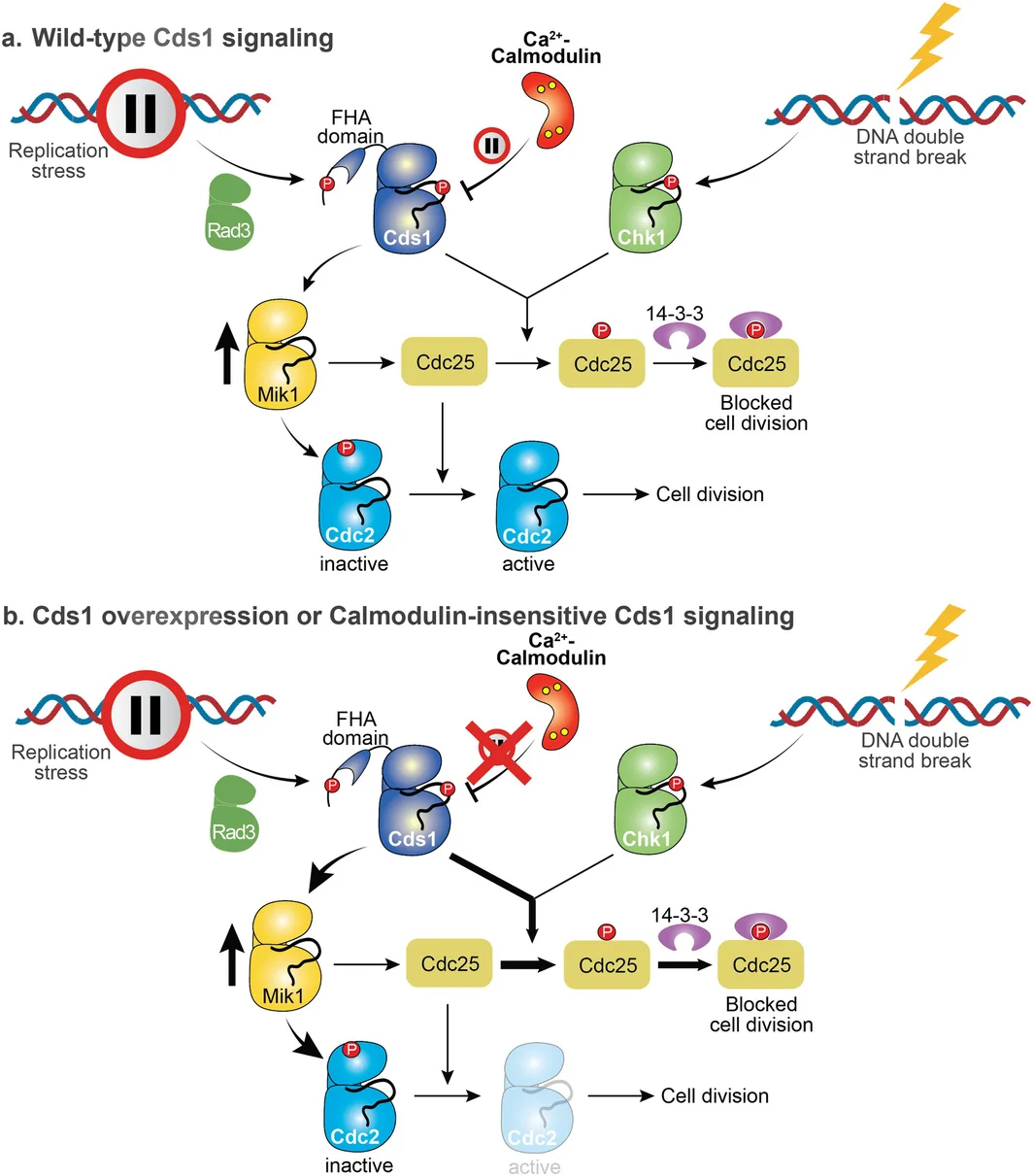
Schematic depicting the crucial role of Cds1 activity in replication checkpoint regulation. (a) In wild‐type *S. pombe*, a delicate balance between Cds1 and Chk1 kinase activities is maintained to pause S phase exit and allow replication and DNA repair to be completed [42]. Cds1 activity is dampened by Calmodulin interaction (this work) ensuring the correct balance of Cds1 and Chk1 activities, such that Cds1 can drive Mik1 expression and its phosphorylation of Cdc2 kinase at Y15 to inhibit Cdc2 activity and suppress cell cycle progression [19]. Simultaneously, Cds1 and Chk1 phosphorylate Cdc25 phosphatase, leading to engagement of 14‐3‐3 and incapacitation by sequestration [21]. (b) Overexpression [18] or compromise of Calmodulin suppression (this work) blocks S phase exit by driving Mik1 expression and/or Wee1 activation and commensurate Cdc2 inhibition, in addition to Cdc25 phosphorylation and sequestration. Figure concept based on ref. [19]; DNA and thunderbolt schematics are from BioRender.

A member of the CAMK family, CHK2, plays an important role in regulating the cell cycle following DNA damage in mammalian cells [14]. Recently, we reported that Ca^2+^‐Calmodulin binds to CHK2, inhibiting phosphorylation of downstream substrates and facilitating cell cycle progression and DNA repair [11]. CHK2 is the human orthologue of Cds1 kinase found in the fission yeast, *Schizosaccharomyces pombe*, which plays a critical role in regulating exit from the replication checkpoint to progress from S to M phase in the cell cycle [17, 18, 19, 20]. Cds1 activation relies on 6 Rad proteins (Rad1, Rad3, Rad9, Rad17, Rad26, and Hus1) that serve essential functions in DNA damage sensing and the replication checkpoint [34, 35]. Rad3 (ATR kinase in humans) and its obligate co‐factor Rad26 (ATRIP in humans) serve a specific role in phosphorylating T11 in the N‐terminal FHA domain of Cds1 following replicative stress exposure [36, 37, 38]. T11 phosphorylation permits the Cds1 FHA domain to bind the phosphorylated T11 site on another Cds1 protomer to form dimers, which can subsequently undergo transphosphorylation on T328 within the activation loop to promote Cds1 activity [39]. Activated Cds1 can then attenuate the transition from S to M phase by promoting expression of the crucial kinase, Mik1, or activity of Wee1 kinase, each of which can phosphorylate Y15 of Cdc2 kinase to dampen its catalytic activity and block cell cycle progression [18, 40, 41]. Simultaneously, Cds1 works in concert with the DNA damage response kinase, Chk1, to phosphorylate key substrates, including the Cdc25 phosphatase [19, 20, 21], to pause cell cycle progression (Figure 4a).

CHK2 and Cds1 are homologues that share a common domain architecture and contain evolutionarily conserved residues that were previously implicated in Calmodulin binding in human CHK2 [11]. Here, we disrupted Calmodulin sensitivity of Cds1 by mutating the counterpart of CHK2 K373 – K317 – in the kinase domain activation loop, resulting in a *S. pombe* phenotype akin to Cds1 overexpression (Figure 4b). Our findings indicate that a Goldilocks level of Cds1 activity is essential for replication checkpoint exit, and that Ca^2+^‐Calmodulin binding serves as an essential brake on Cds1 kinase activity to allow completion of DNA replication and repair prior to S to M cell cycle progression. Just as the site centred on K317 in Cds1 can be targeted by Calmodulin to tune Cds1 activity, the homologous CHK2 residue, K373, is highly sensitive to substitution, consistent with the inhibition serving as a switch to modulate kinase activity. CHK2 K373 substitution to Glu has been reported as recurrent, and possibly causative of cancers owing to compromised CHK2 activity [43]. Whether this is attributable to lost Calmodulin interaction, deficits in activation loop phosphorylation, lost Lys methylation or another impact on protein interactions or post‐translational modifications is unknown but, regardless, speaks to the CHK2 activation loop as a hotspot for modulation of kinase activity.

Ca^2+^‐Calmodulin is best described as a regulator of kinase activity via binding to a sequence C‐terminal to the kinase domain within CAMK family kinases, displacing the autoinhibitory segment from the active site and thereby activating the substrate kinase [44]. However, recent studies of CHK2 [11], PSKH1 [10], BRSK1/2 [13] and eEF‐2K [12] kinases support the idea that Ca^2+^‐Calmodulin can directly bind to and modulate the activity of kinase domains, even in the absence of a conventional Calmodulin‐binding sequence. These studies raise the tantalising prospect that Calmodulin might function more broadly as a regulator of kinases, including within the CAMK family, that lack a conventional Calmodulin‐binding sequence, and illustrate that Calmodulin plasticity allows engagement of kinase domain surfaces rather than solely linear motifs. Furthermore, conservation of Calmodulin suppression of kinase activity in yeast and human Cds1/CHK2 orthologues supports the existence of an ancient mechanism, raising the prospect that Calmodulin and other Ca^2+^ sensor proteins might exert analogous, deeply conserved regulatory functions on protein kinases. Such functions would be expected to couple changes in Ca^2+^ flux to cell cycle progression, capitalising on the role of Ca^2+^ as a second messenger to tune the activities of kinases at the heart of cell cycle signalling. Indeed, Ca^2+^ sensor proteins were recently shown to tune the catalytic activity of PSKH1 kinase, with Calmodulin elevating and Reticulocalbin‐3 suppressing kinase activity [10]. Both Ca^2+^ sensors bind overlapping sites on PSKH1, yet each leads to distinct outputs that are intricately coupled to intracellular Ca^2+^ flux. While Calmodulin binds Ca^2+^ with nanomolar affinity, leading to increased PSKH1 activity at low Ca^2+^ levels, Reticulocalbin‐3 binds Ca^2+^ orders of magnitude less tightly, meaning that Reticulocalbin‐3 can be licensed to suppress PSKH1 activity only in the presence of high Ca^2+^. The precise scope of kinase regulation by Calmodulin and other Ca^2+^ sensors, and whether they enhance or suppress kinase activity, and their impacts on cell signalling remain largely unknown and represent an intriguing avenue for future discovery.

## Funding

This work was supported by the National Health and Medical Research Council, 2034044, 2001817, 2034104. Australian Research Council, DP210102840.

## Conflicts of Interest

The authors declare no conflicts of interest.

## Supporting information


**Supplementary Figure 1:** Purification and intact mass spectrometry (a) Reducing SDS‐PAGE analysis of purified full‐length *S. pombe* Cds1 wild‐type Cds1 K317A and *S. pombe* Calmodulin visualised by stain‐free imaging. Positions of molecular weight standards (first lane) are annotated on the left of the gel. The purity of the shown protein preparations are representative of at least 3 independent expressions and purifications for each construct. Intact mass spectrometry spectra of purified (b) *S. pombe* Calmodulin (expected MW = 17,177 Da), (c) full‐length *S. pombe* Cds1 wild‐type (expected MW = 52,286 Da) and (d) full‐length *S. pombe* Cds1 K317A (expected MW = 52,229 Da). The measured molecular weights for each sample and the presence of any phosphate modifications are annotated.
**Supplementary Figure 2:** AlphaFold3 modelling confidence metrics. AlphaFold model with *S. pombe* Cds1 in complex with *S. pombe* Calmodulin (and four Ca^2+^ ions). ipTM = 0.24; pTM = 0.48. Left hand panel show the per‐residue measure of local confidence score (pLDDT) mapped to a Richardson (ribbon) diagram. The rainbow legend shows the pLDDT confidence score. Right side panel show the Predicted Alignment Error (PAE) plot for the model (in Å).
**Supplementary Figure 3:** Phosphorylated SDA crosslinks of Cds1: Calmodulin. (a) SDA chemical cross‐linking of Cds1 (grey) and Calmodulin (blue) when phosphorylation (pSer, pThr, pTyr) was included as a variable modification for crosslinking analysis. Beige dashed lines represent crosslinks. Phosphorylated amino acids (orange circles with a P) involved in crosslinking matched peptides at Tyr193, Thr 213 and Thr291. (b) AlphaFold ribbon model of the Cds1 kinase domain (grey) and Calmodulin (blue). FHA domain is coloured off‐white. Ca^2+^ ions are shown as yellow spheres. Phosphorylated residues that were crosslinked by SDA (Tyr193, Thr213 and Thr291) are highlighted.

## Data Availability

Materials are available from the corresponding authors under a Materials Transfer Agreement. Mass spectrometry data have been deposited to the ProteomeXchange Consortium via the PRIDE partner repository [45] with the dataset identifier PXD070930.
