# Supplementary material for: An Ancestral Mechanism of Calmodulin Binding to Cds1 Kinase Inhibits Catalytic Activity
Source: IUBMB Life. 2026 Aug 26;78(8):e70128. doi: 10.1002/iub.70128 (PMC13519133; doi:10.1002/iub.70128)

SUPPLEMENTARY INFORMATION

An ancestral mechanism of Calmodulin binding to Cds1 kinase inhibits catalytic activity

Stephanie A. Manovella[1,2], Tingting Wang[3] Samuel N. Young[2], Toby A. Dite[2,4], Vineet Vaibhav[2,4], Steve Binos[2,4], Laura F. Dagley[2,4], Anh T.N. Nguyen[1], Anthony R. Means[5], Janni Petersen[3], John W. Scott[1,6*], James M. Murphy[1,2,4,6*], Christopher R. Horne[1,2,4,6*]

[1]Drug Discovery Biology, Monash Institute of Pharmaceutical Sciences, Monash University, Victoria 3052, Australia

[2]Walter and Eliza Hall Institute of Medical Research, 1G Royal Parade, Parkville, Victoria 3052, Australia

[3]Flinders Health and Medical Research Institute, Flinders University, Adelaide, South Australia 5001, Australia

[4]Department of Medical Biology, University of Melbourne, Parkville, Victoria 3052, Australia

[5]Molecular and Cellular Biology, Baylor College of Medicine, Houston, TX, 77030, USA

[6]These authors contributed equally to the supervision of the study

[*]Please direct correspondence to horne.c@wehi.edu.au (CRH), john.scott@monash.edu (JWS) or jamesm@wehi.edu.au (JMM)

SUPPLEMENTARY FIGURES

Supplementary Figure 1 | Purification and intact mass spectrometry (a) Reducing SDS-PAGE analysis of purified full-length *S. pombe* Cds1 wild-type Cds1 K317A and *S. pombe* Calmodulin visualized by stain-free imaging. Positions of molecular weight standards (first lane) are annotated on the left of the gel. The purity of the shown protein preparations are representative of at least 3 independent expressions and purifications for each construct. Intact mass spectrometry spectra of purified **(b)** *S. pombe* Calmodulin (expected MW = 17177 Da), **(c)** full-length *S. pombe* Cds1 wild-type (expected MW = 52286 Da) and **(d)** full-length *S. pombe* Cds1 K317A (expected MW = 52229 Da). The measured molecular weights for each sample and the presence of any phosphate modifications are annotated.

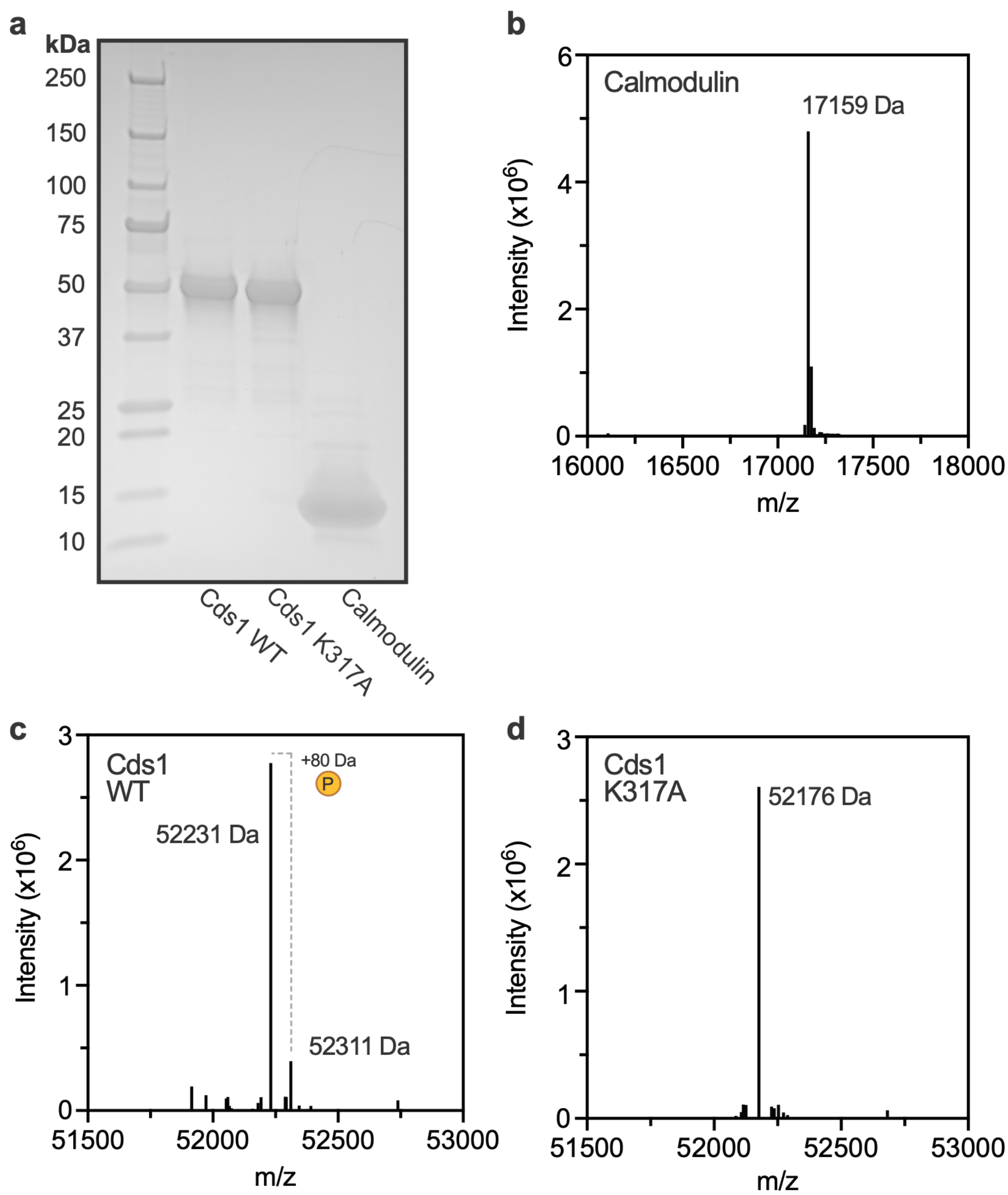

Supplementary Figure 2 | AlphaFold3 modelling confidence metrics. AlphaFold model with *S.pombe* Cds1 in complex with *S.pombe* Calmodulin (and four Ca^{2+} ions). ipTM = 0.24; pTM = 0.48. Left hand panel show the per-residue measure of local confidence score (pLDDT) mapped to a Richardson (ribbon) diagram. The rainbow legend shows the pLDDT confidence score. Right side panel show the Predicted Alignment Error (PAE) plot for the model (in Å).

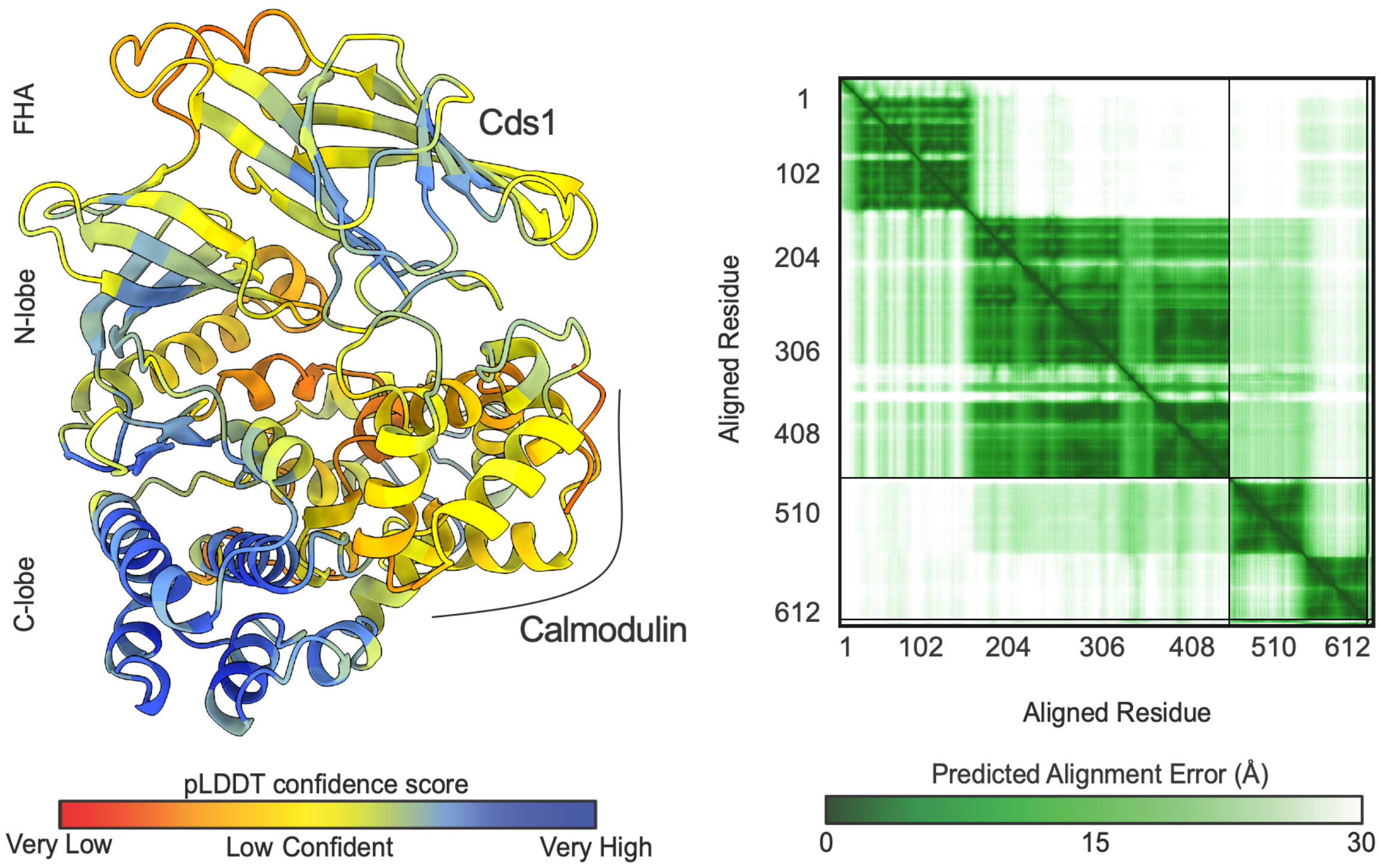

Supplementary Figure 3 | Phosphorylated SDA crosslinks of Cds1: Calmodulin. (a) SDA chemical cross-linking of Cds1 (grey) and Calmodulin (blue) when phosphorylation (pSer, pThr, pTyr) was included as a variable modification for crosslinking analysis. Beige dashed lines represent crosslinks. Phosphorylated amino acids (orange circles with a P) involved in crosslinking matched peptides at Tyr193, Thr 213 and Thr291. **(b)** AlphaFold ribbon model of the Cds1 kinase domain (grey) and Calmodulin (blue). FHA domain is coloured off-white. Ca^{2+} ions are shown as yellow spheres. Phosphorylated residues that were crosslinked by SDA (Tyr193, Thr213 and Thr291) are highlighted.

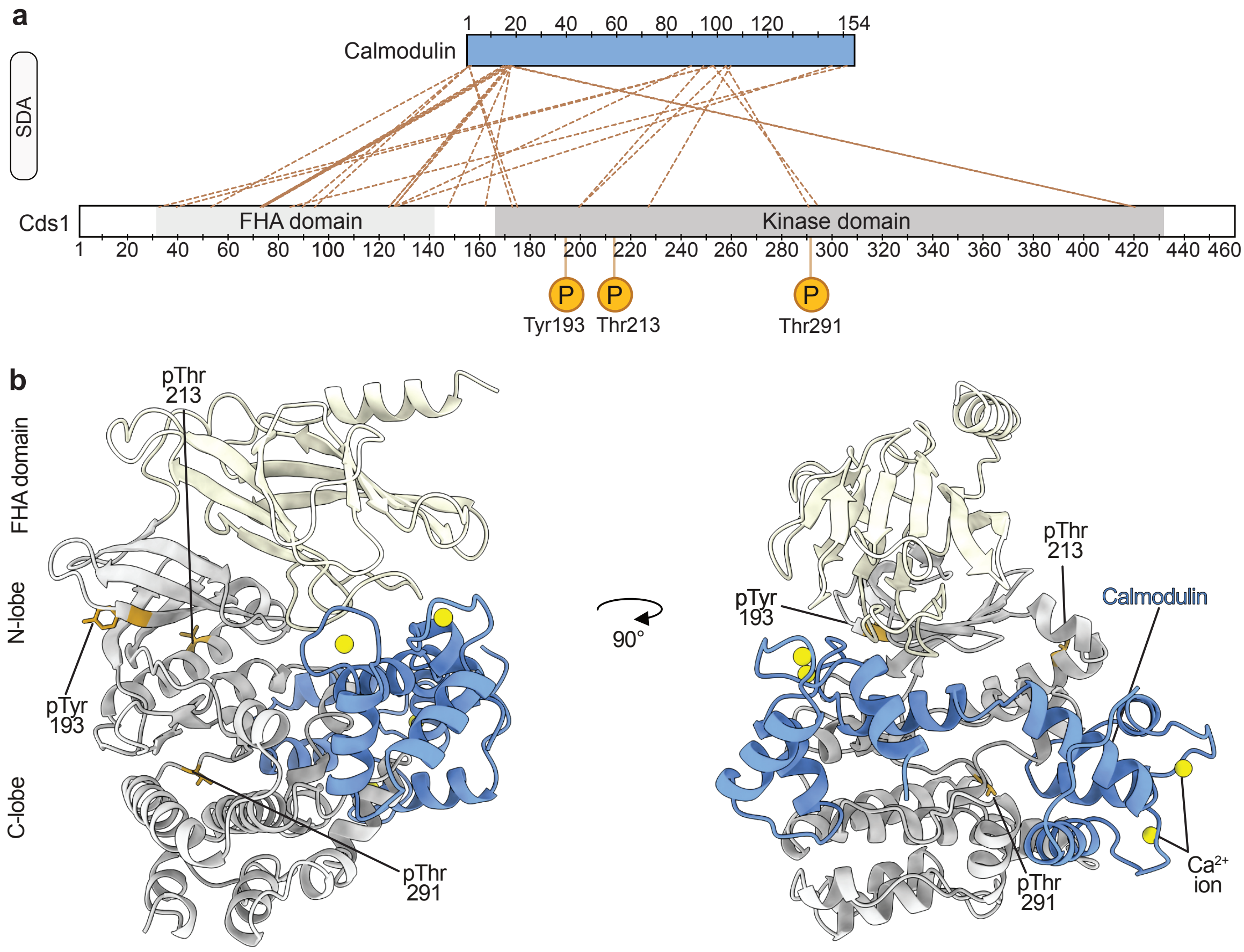

Supplement: Supplementary file 1 [file IUB-78-0-s001.pdf]
